# Protein ensembles link genotype to phenotype

**DOI:** 10.1371/journal.pcbi.1006648

**Published:** 2019-06-20

**Authors:** Ruth Nussinov, Chung-Jung Tsai, Hyunbum Jang

**Affiliations:** 1 Cancer and Inflammation Program, Leidos Biomedical Research, Inc., Frederick National Laboratory for Cancer Research, National Cancer Institute at Frederick, Frederick, Maryland, United States of America; 2 Sackler Institute of Molecular Medicine, Department of Human Genetics and Molecular Medicine, Sackler School of Medicine, Tel Aviv University, Tel Aviv, Israel; Centre National de la Recherche Scientifique, FRANCE

## Abstract

Classically, phenotype is what is observed, and genotype is the genetic makeup. Statistical studies aim to project phenotypic likelihoods of genotypic patterns. The traditional genotype-to-phenotype theory embraces the view that the encoded protein shape together with gene expression level largely determines the resulting phenotypic trait. Here, we point out that the molecular biology revolution at the turn of the century explained that the gene encodes not one but ensembles of conformations, which in turn spell all possible gene-associated phenotypes. The significance of a dynamic ensemble view is in understanding the linkage between genetic change and the gained observable physical or biochemical characteristics. Thus, despite the transformative shift in our understanding of the basis of protein structure and function, the literature still commonly relates to the classical genotype–phenotype paradigm. This is important because an ensemble view clarifies how even seemingly small genetic alterations can lead to pleiotropic traits in adaptive evolution and in disease, why cellular pathways can be modified in monogenic and polygenic traits, and how the environment may tweak protein function.

## Introduction

The terms genotype and phenotype have been in use at least since the turn of the last century. Genotype has been defined as the genetic makeup of an organism or of a specific characteristic. Phenotype (from Greek phainein, meaning “to show,” and typos, meaning “type”) has been construed as the composite of the organism’s observable characteristics or traits, such as morphology, development, biochemical, and physiological properties. Classically, the genotype of an organism has been described as the inherited genetic material coding for all processes in the organism’s life. It provided some measurement of how an individual is specialized within a species based on its genomic sequence. By contrast, the phenotype referred to the observation that similar genotypes can differ in their expression under different environmental and developmental conditions. Typically, an individual’s genotype relates to a particular gene of interest or to the combination of alleles that the individual organism or cell carries. To explain how the genotype determines the phenotype, population genetics [1] pointed out that (1) in real populations, phenotypic ratios are determined by the frequency of alleles in the population as well as by whether the alleles are in dominant or recessive form, (2) the number of phenotypes produced for a given trait depends on how many genes control that trait, and (3) there is no one-to-one mapping between genes and traits. Exactly what is a “trait” was not well defined.

**Fig 1:**
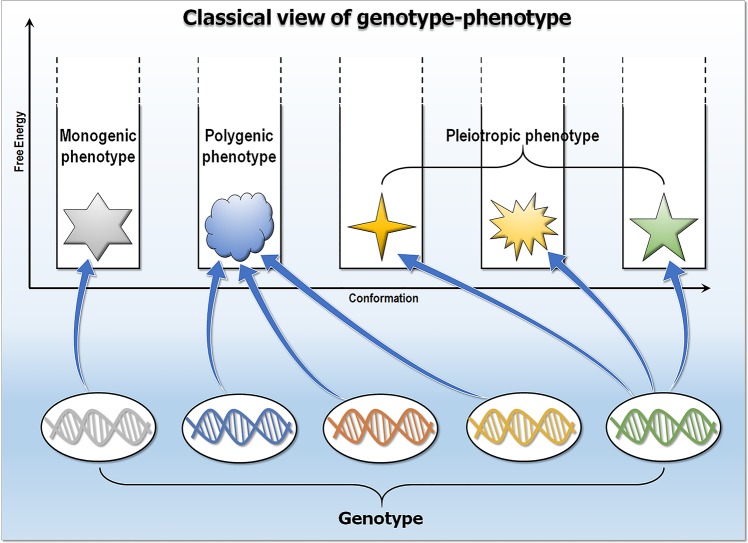
Classical view of genotype–phenotype. In this view, a protein or the gene product is considered to have one shape with a single function. Monogenic traits are expressed by single genes, whereas polygenic traits are affected by multiple genes. Seemingly unrelated phenotypic traits are pleiotropy that can be expressed by a single gene.

The classical genotype–phenotype interpretation dates to a period when a protein, the gene product, was believed to exist in one shape with a single function (Fig 1). Evolution was perceived to optimize that shape for this function. Phenotype was considered as a visually observable property. Over a century later, with the understanding of the basis of protein structure and function having undergone a dramatic revolution, the genotype–phenotype paradigm remains unchanged. Scientific publications still commonly relate to it in terms of this weathered image. This view overlooks the fact that biomolecules exist as heterogeneous dynamic interconverting states with varying energies, and the multiple traits may mirror those protein states. The “second molecular biology revolution” [2], which imported newer concepts from physics and chemistry to molecular biology, such as the powerful idea of the free energy landscape [3], allows a new view of this genotype–phenotype dogma. Biomolecules should be thought of not as static single shapes but as statistical ensembles [4–7]. Here, we explain that structural ensembles—which allow proteins to fulfill their functions—link genotypes to phenotypes. Thus, within the broad cellular context, it is the network that controls transcription via gene regulation [8–30]; here, however, we relate to mutations that affect function at the lower, protein level.

## Protein evolution in terms of biophysics

The evolution of proteins in terms of their conformational ensembles has not been overlooked [31, 32]. In a series of studies, the relationship of the protein’s structure and conformational dynamics to its function and thus its fitness has been explored, not through classical phylogenetic approaches, which largely overlook biophysical principles, but by evaluating how mutations impact protein structure, which already has marginal stability [33–35]. Mutations can also shift the equilibrium from an inactive, autoinhibited state to the active state, as for example observed in Raf and phosphoinositide 3-kinase (PI3K) [36]. A linkage between evolution and biophysics was also explored by changes in dynamic flexibility profiles [37], by protein interaction networks [38], by protein adaptation as observed by the functional impact of multiple mutations, by identifying key adaptive mutational solutions to the same selective pressure [39, 40], by extant fold-switching proteins [41], and by exploring the relationship between metastability, the fitness landscape, and sequence divergence [32]. Evolutionary selection has been explored in terms of the dynamics of structural evolution [42], and evidence for evolutionary selection in cotranslational folding was also found [43]. The stability of a viral protein was observed to correlate with its evolutionary dynamics [44], and the evolution of the biophysical fitness landscape of an RNA virus was explored as well [45]. Even a web app has recently been set up for such exploration [46]. Comprehensive analysis indicated that evolution conserves functional dynamic motions; clusters of conserved residues have a signature characteristic of protein domains, being spatially separated but individually compact [47]; and sequence and structurally conserved residues tend have a lower fluctuation than other residues [48]. Some studies emphasized the challenge of forecasting mutational outcomes, arguing that ensembles make evolution unpredictable [49].

Can the biophysical view of conformational ensembles link to classical evolutional concepts, such as phenotypic plasticity, bet-hedging, canalization, and assimilation? Classically, a genotype’s phenotype is viewed as relatively invariant, irrespective of different environments [50]. However, recently, a linkage was proposed via a “plasticity-first evolution” hypothesis that suggested how phenotypic plasticity may have facilitated macroevolutionary change [51]. Protein conformational diversity was suggested to correlate with evolutionary rate [52], and “phenotypic plasticity” through nongenetic heterogeneity was recently proposed to be driven by protein conformational dynamics [53]. The authors argued that mutations alter the relative probabilities of conformations, thus changing the effects of future mutations, resulting in uncertainty in the effect of each subsequent mutation and consequently prediction. Coincidentally, large-scale analysis has further shown that mutational effects on the conformations may also be small, even smaller than among proteins of identical sequences [54]. Finally, recent reviews described bridging the physical scales in evolutionary biology, from protein sequence space to fitness of organisms and populations [55], delineating the evolution of function from such a perspective [56].

The conformational space is vast, with the available X-crystal structures covering only a certain fraction, as recently elegantly documented for the Abl tyrosine kinase, for which molecular dynamics simulations and Markov state models identified a protein conformation apparently in a Lilly in-house structure of Abl with WHI-P15 but not in the Protein Data Bank (PDB) [57]. Even though here exploited for drug discovery, it is reasonable to expect that evolution has made use of such vast ensembles as well, adapting them in different ways [58, 59], including in thermostability [60, 61], diverse cellular environments [62], protein disorder and the switches between the ordered and the disordered state [63, 64], detailed linker histone sequence and posttranslational modification (PTM) [65] as well as catalysis of an (O-linked β-N-acetylglucosamine) O-GlcNAc PTM of nuclear and cytosolic protein [66], allosteric interaction networks and signaling pathways [67], and in higher-order organization [68]. This expectation not only can help in prediction of ligand binding [69] but also has inspired the proposition that accounting for conformational heterogeneity and dynamics would benefit protein design methods [70]. Along these lines, recent reviews underscored the role of conformational dynamics in enzyme evolution toward new functions and suggested how conformational dynamism can be exploited in computational enzyme design protocols [71] and how a priori knowledge of an allosteric network could improve design through navigation of the design space [72]. Thus, biophysics is not concerned with quantitative association of gene loci nor with certain statistical measurements that are hallmarks of classical evolution; it views evolution at the basic conformational level and considers protein sequence and structural space and fitness of organisms and populations.

## Statistical relationships between genotype and phenotype

**Fig 2:**
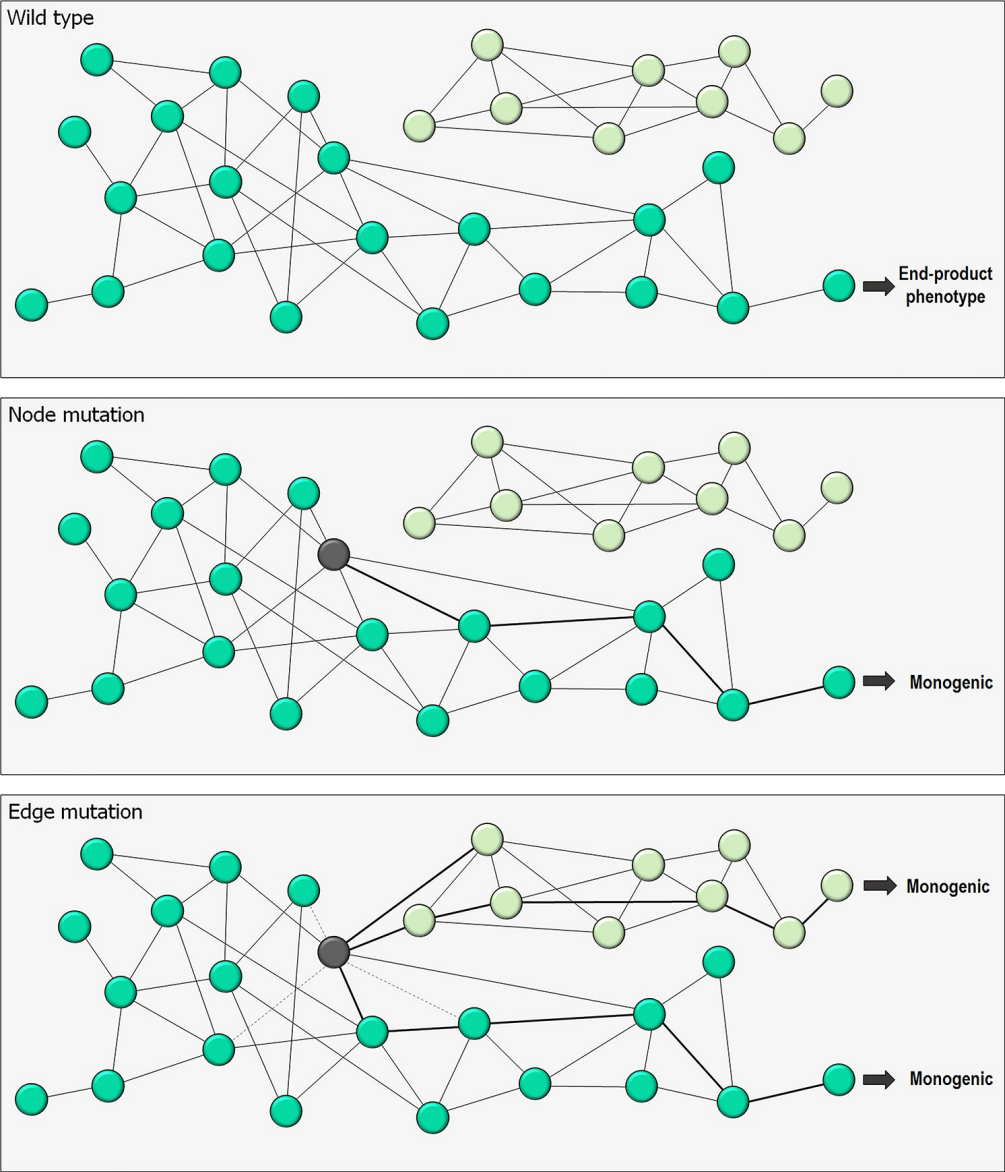
Network perturbations of genotype–phenotype. Mutations can shift phenotype traits generated from wild-type trait (upper panel), affecting solely the protein (a “node”) or protein–protein interaction (an “edge”). The former and latter are node and edge mutations, respectively. The node mutation (middle panel) generates a monogenetic trait, whereas the edge mutation (bottom panel) creates or breaks the protein–protein interactions yielding monogenetic or polygenetic traits. An example shown for edge trait is polygenetic.

Monogenic traits are affected by single genes, polygenic traits are affected by multiple genes, and pleiotropy occurs when one gene influences multiple, seemingly unrelated phenotypic traits [73–75] (Fig 1). Quantitative association of trait loci (QTL) aims to explain the genetic basis of variation in complex traits by linking phenotype data (trait measurements) to genotype data (e.g., single-nucleotide polymorphisms [SNPs]). However, quantifying traits is challenging; matters of contention include traits’ definitions, interdependence, and selection. Statistics suggest that the frequency of pleiotropy is not high. Instead, the average phenotypic effect of a mutation on a trait increases with the number of traits that are affected by the mutation. Wagner and Zhang pointed out that pleiotropy may result from multiple molecular functions of a gene (type I pleiotropy) or from multiple outcomes of a single molecular function (type II pleiotropy) and that type II is the most common, which is why developing drugs that target only one particular phenotype of a pleiotropic gene may fail [74]. Here, we consider two types of scenarios (Fig 2). In the first, the mutations affect solely the protein (a “node”). This scenario applies only to monogenic phenotypes. In the second, the mutations create or break a protein–protein interaction (an “edge”). This scenario works by altering the cellular network. It can take place in gained monogenic or polygenic phenotypes. Since we focus on protein ensembles, we only consider mutations/SNPs in protein-coding regions.

Genome-wide association studies (GWAS) seek relationships between sites of common genome sequence variation and disease predisposition. They have already revealed the genetic basis of over 50 disease-susceptibility loci and provided insight into the allelic architecture of multifactorial traits [75], indicating that the approach can successfully identify common SNP variants with sufficiently large phenotypic change and illuminate relationships between changes in genome sequence and phenotypic variation. Challenges are not so much in detecting the statistical association signals but in relating them to the molecular mechanisms of phenotypic expression. There are 20,000 to 30,000 genes in the human body; there are millions of phenotypic traits [76]. Statistical studies such as those above can reveal relations and associations; they are unable to explain how SNPs, disease-related mutations, or mutations in adaptive evolution lead to phenotypic change. Since each record contains many variables, it is difficult to fully interpret the observed statistical biases [77]. This, however, becomes possible when we consider gene products as ensembles of dynamic, interconverting states. The linkage between genotype and phenotype can be understood in terms of the statistics of the ensemble.

## The genotype encodes a conformational ensemble

**Fig 3:**
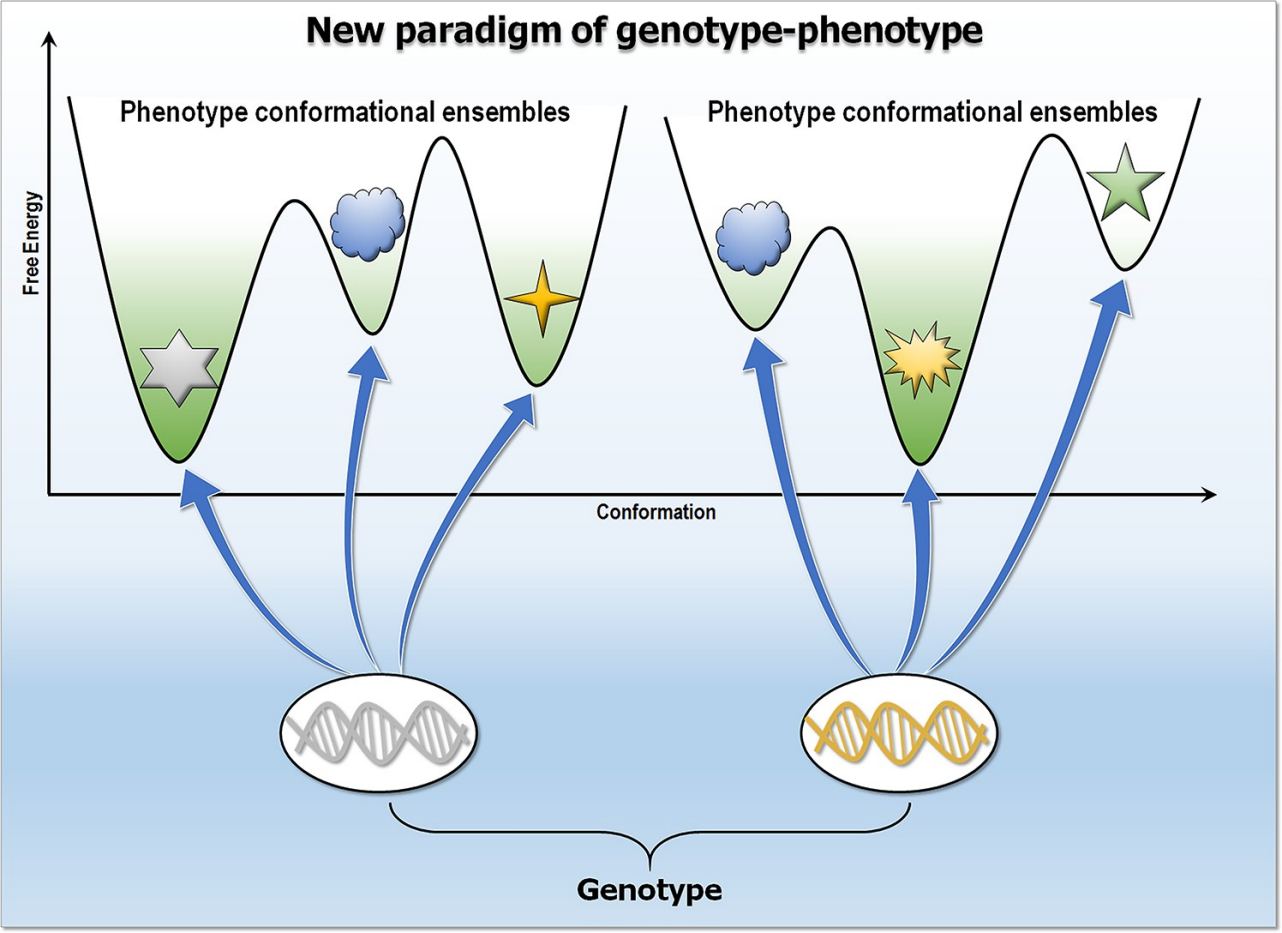
New paradigm of genotype–phenotype. In this view, genotype encodes a distinct conformational ensemble in all states. Populations determine the specific phenotype traits that link to genotype. Mutations shift the equilibrium of preexisting conformational ensembles altering phenotypes.

In line with the rich biophysics literature, with some of the most recent cited above (as well as [34, 78]), and the long-standing awareness that multiple/promiscuous function drives transitions [35, 79], the genotype should be thought of not as determining a structure but instead as establishing a distinct conformational ensemble, which in turn specifies the phenotype (Fig 3). This ensemble embodies all states, including the orthosteric ligand-bound conformation, the activated (or inactivated) allosteric modulated states, posttranslationally modified states, transition states, and nonfunctional states serving as a reservoir for emerging functions [4–7]. The ensemble can be described by statistical mechanical laws, and its populations follow statistical distributions. Their specific distributions reflect the environment and conditions of the protein’s milieu. The “environment and conditions” include both the physical surrounding, such as concentration, pH [80], presence of solutes or lipids, ions (where binding of sodium ions was shown to shift the population toward conformational states [81]) and covalent changes in the protein, such as mutations and their combinations, and phosphorylation, ubiquitylation, etc. A shift of the conformational ensemble that alters the highly populated state may define the phenotype. This thesis observes that evolution encoded all states and their populations in the genotype and optimized them, including rare states, for distinct phenotypes.

The genotype—i.e., the genetic makeup of cells—and the phenotype have been in the limelight for decades. The genotype and phenotype have been linked through expression of the specific encoded proteins. The linkage has been understood in terms of the specific three-dimensional structures that they obtain. However, the genetic makeup is expressed through ensembles of states and their interactions, and it is the distinct states in the ensemble that define the phenotype. A specific function is executed by a specific conformation, and a switch between the states, typically elicited by an allosteric event, can lead to a phenotypic change [82–86]. Examples include the G protein–coupled receptors (GPCRs), highly dynamic proteins that adopt multiple active states linked to distinct functional outcomes [87].

## Structural ensembles link genotype to phenotype

Sickle cell anemia—a disorder that leads to atypical hemoglobin molecules that can distort red blood cells into a sickle, crescent shape resulting in anemia, repeated infections, and periodic episodes of pain—is one classical monogenic adaptive evolution example. It is a consequence of a single nucleotide/amino acid Glu → Val change. The conformational landscape of hemoglobin encompasses both states; however, the sickle cell conformation is visited more often in the mutant. A more recent example concerns melanocortin receptor 1 (MC1R), which plays a central role in regulation of animal coat color formation. Two nonsynonymous mutations in the *MC1R* gene previously associated with coat color in Chinese Minxian black-fur sheep are not present in the white coat (large-tailed Han) sheep [88]. A striking example concerns the coat color of deer mice [89]. Light coat color provides a selective advantage against visually hunting predators for mice that have recently colonized the light-colored soil of the Nebraska Sand Hills, in contrast to dark-colored deer mice inhabiting nearby dark soils. The phenotype is composed of five color traits: dorsal hue, dorsal brightness, ventral color, dorsal–ventral boundary, and tail stripe. Each trait is statistically associated with a unique set of SNPs, with one exception, a serine deletion in exon 2 that was linked to both ventral color and tail stripe. Each color trait indicates selection. This example suggests (1) that the large-locus effect divides into small- to moderate-effect mutations and (2) that when one gene influences multiple, seemingly unrelated phenotypic traits, it is the individual mutations that bring the population closer to its phenotypic optimum. Thus, small minimally pleiotropic mutations occurring within a single gene may rapidly adapt an organism to multiple phenotypes. In the language of the free energy landscape, a set of mutations provides a complex conformational behavior; however, their combinations can more readily achieve specific optimal conformations. Similar strategies have been adopted by evolution through combinatorial sets of allosteric PTMs, which create recognition barcodes [90]. Each set of SNPs results in one type of coat color through distinct conformations.

A similar complex conformational behavior appears to be involved between the human MC1R pigmentation and skin cancer gene and youthful facial looks. Loss-of-function polymorphisms (multiple functions of a single gene) in human MC1R result in the yellow to reddish pheomelanin, which has a weaker ultraviolet (UV) shielding capacity. Recent analysis of over 8 million SNPs in 2,693 elderly Dutch Europeans indicated that MC1R SNP variants are most significantly associated with perceived facial age, with the homozygote MC1R risk haplotype looking up to 2 years older on average than noncarriers [91]. This association was independent of age, sex, skin color, and sun damage, such as wrinkling and pigmented spots, suggesting independence from melanin synthesis. The strongest associations with perceived facial age were for multiple SNPs; however, it was enhanced for four SNPs—variants rs1805005 (V60L), rs1805007 (R151C), rs1805008 (R160W), and rs1805009 (D294H)—known to be missense loss-of-function variants [92], causing phenotypes such as red hair color and pale skin [92, 93], and involved in age-related skin phenotypes such as pigmented spots [94]. Along similar lines, across a total of 2,459 patients at different ages and 53 families, the presence of a mutation in a sarcomere gene (cardiac myosin-binding protein C gene [*MYBPC3*] and myosin heavy chain 7 gene [*MYH7*]) in familial hypertrophic cardiomyopathy (HCM) has been associated with a number of phenotypic clinical features, including age at presentation, gender, family history of familial HCM and sudden cardiac death (SCD), and maximum left ventricular wall thickness (MLVWT) [95].

An additional combinatorial conformational barcodes example concerns *MYH9*-related disorders, a group of rare autosomal dominant platelet disorders caused by mutations in the *MYH9* gene encoding the nonmuscle myosin heavy chain II-A (NMMHC-IIA). Nonsyndromic forms are characterized by macrothrombocytopenia with giant platelets and leukocyte inclusion bodies; syndromic forms combine these hematological features with deafness and/or nephropathy and/or cataracts. A recent 8-year study of a large cohort of 109 patients from 37 sporadic cases and 39 unrelated families identified 43 genetic variants. Thirty-three of these were missense mutations. Distinct combinations of this heterogeneous mutational landscape resulted in specific disease phenotype [96].

Finally, a broad, systematic study of genotype–phenotype relationships mapped thousands of missense point mutations and in-frame insertions and deletions related to disease. Specific locations of distinct mutations of the same gene on the interface related to disease specificity. This was experimentally validated for the *MLH1*–*PMS2*, Wiskott–Aldrich syndrome protein (*WASP*)–cell division control protein 42 (*CDC42*), and tumor protein (*TP*) *63*–*TP73* interactions [97]. Mutations in protein–protein interfaces (edges) that change the conformational distributions can lead to a selection of a different binding partner, thereby leading to a phenotype change through altered interactions.

## Single mutations

Above, sets of nonsense mutations combinatorially decide the preferred conformational states that provoke the phenotype. Below, we give examples of single-point mutations also acting by shifting the conformational landscape. Although in such cases it has been generally assumed that the phenotype is determined by a single (“driver”) substitution, additional—albeit to date mostly unidentified—previous or subsequent mutational events may cooperate. Low-frequency presumed “passenger” mutations may act combinatorially with the driver in promoting distinct phenotypic expression [77, 98]. Somatic mutations evolve and accumulate over time and may be expected to affect the distributions of the conformational states, similar to the combinatorial examples above.

Plasma-membrane integrin αIIbβ3 is a major receptor in platelets during clotting. The L33P mutation in knockin mice reduces bleeding and clotting times and elevates the in vivo thrombosis phenotype, platelet attachment, and spreading onto fibrinogen [99]. Under unstimulated conditions, the mutation primes αIIbβ3 intracellular domains for outside-in signaling, increasing Src phosphorylation through talin interactions with the β3 cytoplasmic domain, leading to hypercoagulability and increased risk for coronary thrombosis and stroke. The mutation, resulting in a Pro32-Pro33 sequence, modifies the αIIbβ3 conformational equilibrium. The proline at position 33 was suggested to alter the flexibility in the β3 knee defined by the plexin-semaphorin-integrin (PSI), insulin-like growth factor 1 (IGF-1), and IGF-2 integrin αIIbβ3 extracellular domains, resulting in increased adhesion capacity of human platelet antigens (HPA)-1b platelets to fibrinogen [100]. Stormorken syndrome—a rare autosomal dominant disorder characterized by a phenotype that includes meiosis, thrombocytopenia/thrombocytopathy with bleeding time diathesis, intellectual disability, mild hypocalcemia, muscle fatigue, asplenia, and ichthyosis—provides a second example. The syndrome apparently results from a single gene defect, consistent with mendelian dominant inheritance. Stromal interaction molecule 1 (STIM1) protein mutation p.R304W is observed in patients but not in their unaffected family members. The STIM1 protein is an endoplasmic reticulum (ER) Ca^2+^ sensor. Data suggest that the STIM1 p.R304W mutation shifts the equilibrium toward an altered conformation of the inhibitory helix, unlocking the inhibitory state of STIM1. The mutation causes a gain of function increasing both resting Ca^2+^ levels and store-operated calcium entry [101]. An additional example of a mutation shifting the ensemble relates to the Niemann-Pick disease type C, a fatal neurodegenerative disease. Its major cause is mutations in the Niemann-Pick disease, type C1 (*NPC1*) gene, which encodes a late endosomal polytopic membrane protein required for intracellular cholesterol trafficking. One prevalent mutation (I1061T) causes a folding defect, which results in failure of endosomal localization of the protein, leading to loss-of-function phenotype [102].

## Structural ensemble can link genotype to phenotype through phosphorylation

Hepatitis C virus (HCV) requires only 10 proteins for evading the immune system. Phosphorylation of the intrinsically disordered domain (IDD) of nonstructural protein 5A (NS5A), which is important for replication, changes its dynamics and represents a strategy to expand the viral proteome while limiting its coding capacity. The phosphorylation site is at Thr2332, near one of its polyproline-II motifs. Phosphorylation shifts the conformational ensemble of the NS5A IDD to a state that permitted detection of the polyproline motif by using ^15^N-, ^13^C-based multidimensional NMR spectroscopy. Mutating Thr2332 to alanine in HCV genotype 1b reduced the steady-state level of RNA by 10-fold; this change was lethal for genotype 2a. The lethal phenotype could be rescued by changing Thr2332 to glutamic acid, a phosphomimetic substitution. The inability to produce pT2332-NS5A caused loss of integrity of the virus-induced membranous web/replication organelle [103]. The protein kinase A (PKA)-phosphorylated form of NS5A populates a conformation distinct from that of the unphosphorylated protein. The shifted distribution of the conformational ensemble encoded by the viral genome links HCV genotype to its phenotype, in this case via PTM of intrinsically disordered viral proteins.

## The complexity of the genotype–Phenotype relationship

**Fig 4:**
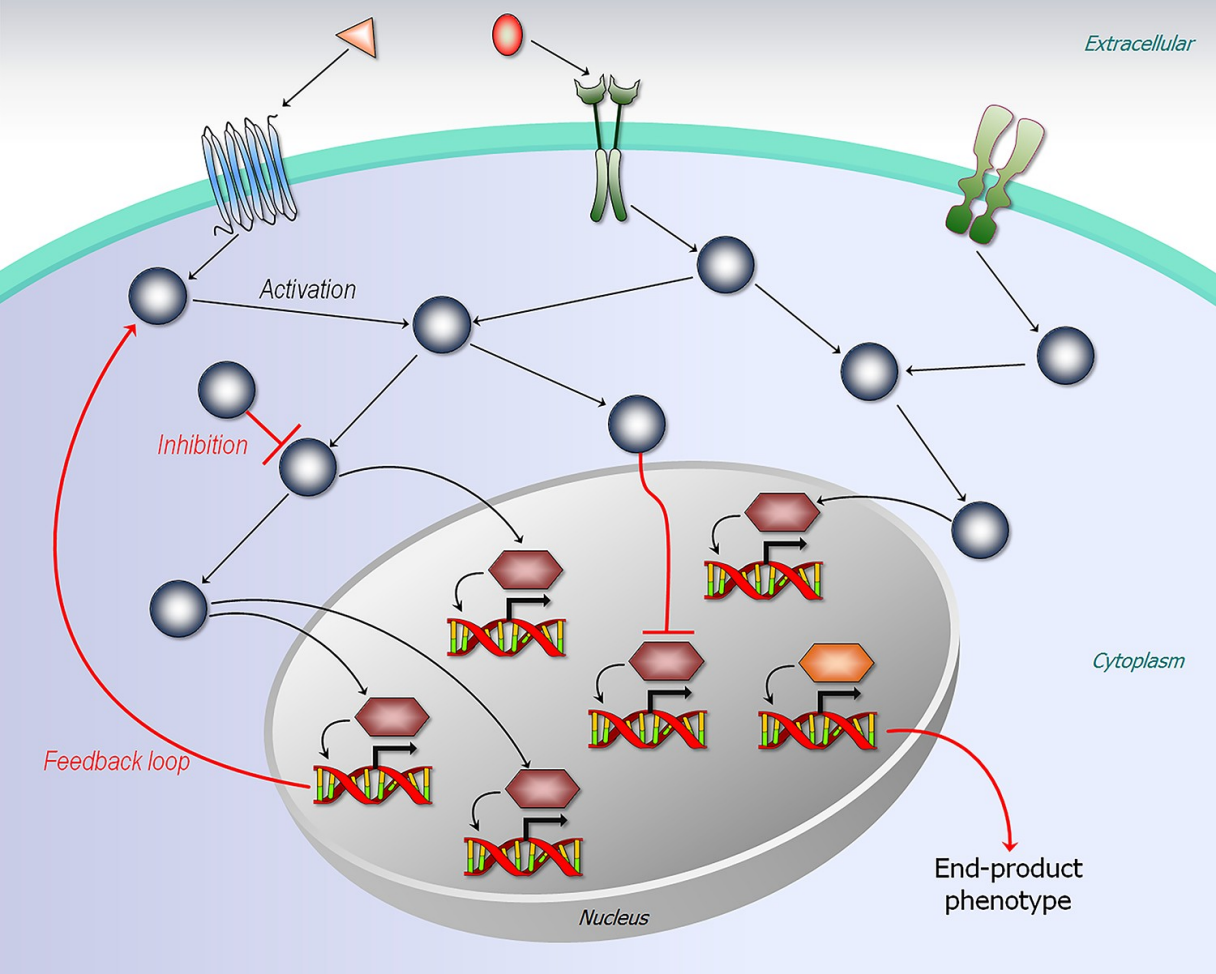
Cellular network. The network controls the transcriptional regulation for gene. Edge mutations can alter the cellular network, expressing the end-product phenotype.

The association between genotype and phenotype is hard to understand. A structural view can help to illuminate the genotype/phenotype landscape. We distinguish between traits expressed by the protein itself (node traits) and by its interactions (edge traits) (Fig 2). Node traits are monogenic and can be considered to lie at the “end” of the pathway. The pigments in the deer mouse provide a good example. Edge traits can be monogenic or polygenic. Self-assembly of sickle cell hemoglobin—a monogenic adaptive evolution trait—creates new edges resulting in polymers. Cancer, a growth and proliferation disease, involves driver mutations in more than one protein; thus, polygenic traits involve the cellular network (Fig 4). For example, mutations in KRas, one of the most highly oncogenic proteins, are often coupled with mutations in Ras effectors [104] or with parallel pathways (Hippo/Yap1) [105, 106]. Oncogenic Ras does not rely on epidermal growth factor receptor (EGFR) signaling—that is, on an EGFR–Ras edge. Raf mutations can work by relieving the need for Ras-driven Raf side-by-side dimerization, which is required for activation [107, 108]. p53 mutations are similarly typically coupled with additional driver mutations in other proteins [109, 110]. Node mutations, such as the pigment examples above, affect only the node; edge mutations (e.g., the disease examples above), which create or break interactions (Fig 2), alter the cellular network [111–116] (Fig 4). All traits—node, edge, and type I (multiple molecular functions of a gene) and the more common type II (multiple consequences of a single molecular function) pleiotropy [74]—can be understood in terms of statistics of protein ensembles (Fig 3). The shift in the populations of the sickle cell hemoglobin following the Glu → Val mutation favors polymerization, similar to the shift in Alzheimer amyloid β (Aβ) mutants, there resulting in amyloid formation [117, 118]. Likewise, edge formation/removal in oncogenic mutants arises from a redistribution of the conformational ensemble [119]. Because distinct mutations can shift the ensemble in different ways, the resulting edges can precipitate altered types of cancer by the same protein, as in the case of KRas driver mutations, which have different frequencies in distinct cancers [120]. This description holds both for adaptive evolution and for disease. The usefulness of a structural ensemble, node-versus-edge view can be seen in its drug targeting implications, as well as in prediction of phenotypes from genotypes.

## Prediction of phenotype from genotype

Phenotypes may involve multiple traits that emerge from multiple nodes and edges, making it difficult to predict and relate specific nodes (proteins or genes) to specific traits [10, 13, 121]; however, networks of pairs of nodes and edges that drive phenotypes can be identified [122, 123]. To accomplish this goal, a network (or pathway)-based approach is adopted: instead of directly connecting genotype with phenotype, genotype variants are assembled into gene networks and subnetworks that are statistically connected to the phenotype are identified [10].

Cell signaling and phenotypic expression take place across time and space and are on length scales from nanometers to micrometers [124], which require consideration of how the genetic variation would affect the function in the cell. Recently, this has led to a strategy based on knowledge of cellular subsystems and their hierarchical organization as defined by the Gene Ontology (GO) or inferred from published datasets [10]. Genotype data are formulated hierarchically in terms of the consequences of the genetic variation at multiple cellular scales. The resulting “ontotype” is interpreted by logical rules. As an example, it was used to predict yeast cell growth phenotype of two new screens of double gene knockouts affecting DNA repair or nuclear lumen.

## Observed conformational changes

**Fig 5:**
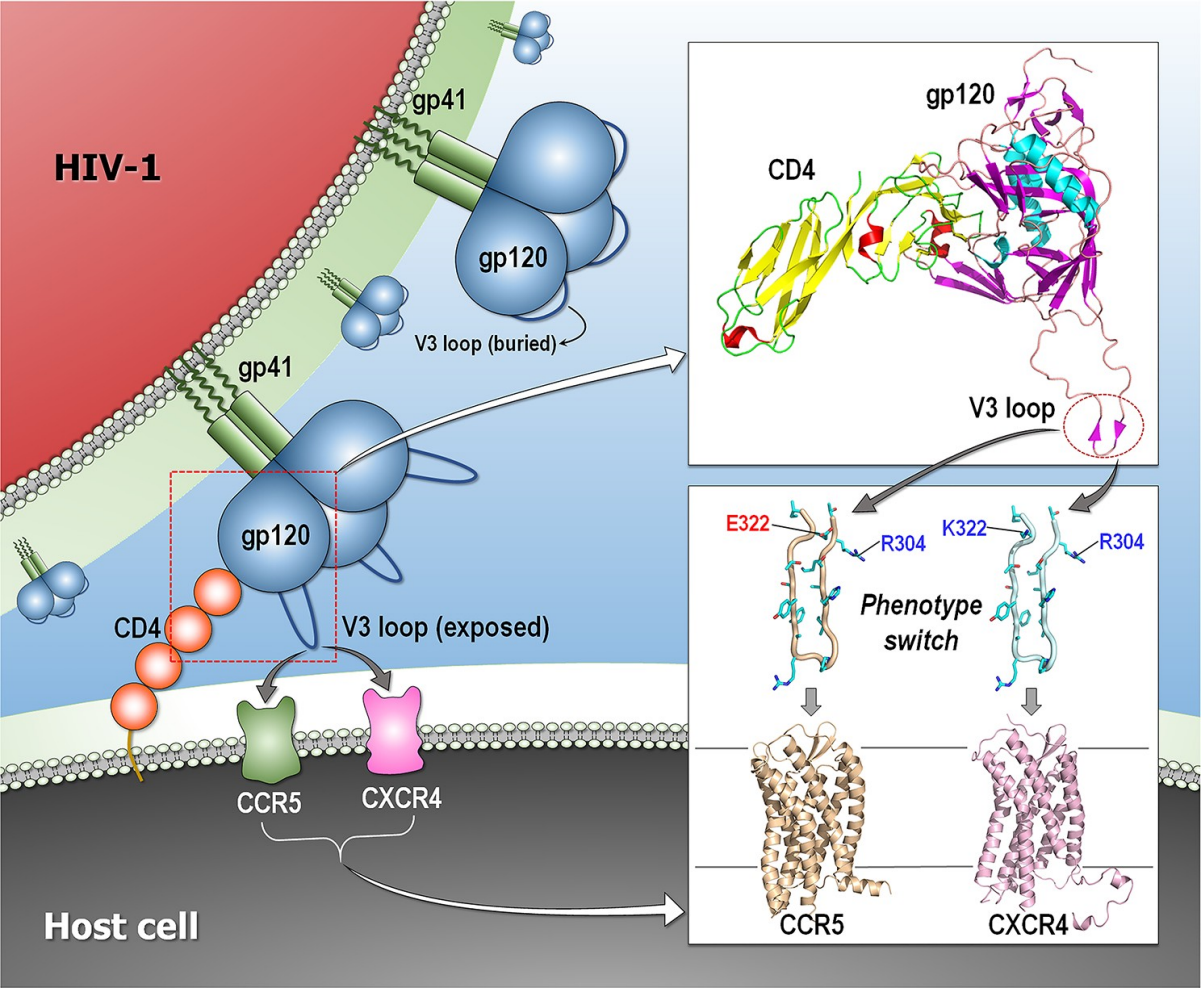
Phenotype switch of HIV-1 entry. Schematic diagram representing the initial process of the HIV-1 entry that led to fusion between the viral and the host cell membranes. The gp120 trimer undergoes a conformational change upon binding to cellular receptor CD4, exposing the variable loop V3. The V3 loop binds to the coreceptor (CCR5 and CXCR4), triggering the entry process. The wild-type V3 loop with positively and negatively charged residues at positions 304 and 322, respectively, recognizes CCR5. A phonotype switch by a mutation from a negatively to a positively charged residue at position 322 in the V3 loop alters coreceptor recognition to CXCR4. Modeled structures are the crystal structures of gp120 (PDB code: 24BC), CCR5 (PDB code: 4MBS), and CXCR4 (PDB code: 3ODU) and the NMR structure of V3 loop (PDB code: 2ESX). CCR5, C-C chemokine receptor type 5; CXCR4, C-X-C chemokine receptor type 4; PDB, Protein Data Bank.

To most clearly demonstrate our thesis, the examples should concomitantly (1) relate to signaling (i.e., pathways with nodes and edges), (2) present a clear visual phenotype (like the fur colors of deer mice or the aging of facial features), and (3) indicate a clear conformational change. We were unable to find such examples. The examples above imply conformational change; but they do not directly show it. Here, we present two examples indicating conformational change; however, they relate to pathogens—thus, no signaling pathway—and the phenotype they confer is disease. The first involves HIV-1 coreceptors (CCR5 and CXCR4) that are critical for virus tropism and pathogenesis (Fig 5). During infection, a mutation from a negatively to a positively charged residue at position 322 in the V3 loop of the HIV-1 envelope glycoprotein gp120 can accomplish a phenotype switch of R5 virus to an X4 virus, and this correlates with disease progression. The NMR structure of the V3 region of an R5 strain illustrates that positively charged and negatively charged residues at positions 304 and 322, respectively, oppose each other in the β-hairpin structure, resulting in stabilizing the R5 conformation. By contrast, in the X4 conformation, electrostatic repulsion between residues 304 and 322 induces a shift in the N-terminal strand, pointing to electrostatic interactions as modulating the conformation and thereby the phenotype switch [125]. The second example involves the classical phenomenon of yeast prion strain variants [126]. Infectious prion states—each of which has distinct conformation—cause distinguishable phenotypes. Even though the spectrum of conformations is identical to that of the noninfectious state, the relative populations differ. Solution NMR, amide hydrogen/deuterium (H/D) exchange, and mutagenesis of two strain conformations, termed Sc4 and Sc37, of the yeast Sup35 prion indicated an overlapping amyloid core composed of tightly packed β-sheets. This stable core is expanded in the Sc37 conformation, explaining why this strain has higher fiber stability, which impedes chaperone-mediated replication. The large conformational differences among prion strains provide a structural basis for their physiological phenotypic behavior.

## Methods to associate ensembles and function

The fact that a protein-coding gene encodes not a single conformation but an ensemble of conformations is indisputable, as is the fact that this must play a role in the making of the phenotype(s). However, the questions of how to capture the multiple states and relate them to distinct functions are challenging. Until recently, X-ray crystallography could only capture a single conformation. Recently, time-resolved crystallography [127–134], diffuse X-ray scattering [135], and cryo-electron microscopy [136–138] have been shown capable of capturing multiple conformations at high resolution along time. These can provide unparalleled insight into catalytic steps in enzyme reactions and binding/dissociations of proteins with their ligands, such as the dissociation of carbon monoxide from myoglobin. However, to date, high-resolution experimental methods linking specific macromolecular states to distinct functions are still challenging. To date, comparisons of available static crystal structures of a given protein complexed with different biological ligands relating to specific functions appear as the method of choice to attain this aim. Computations coupled with lower experimental resolutions have, however, made significant strides toward accomplishing this goal [139, 140], as well as other combined approaches or alone [141–144]. The heterogeneity of molecules is vast, the distinct mechanism of each molecule is often unknown, the sample size of each molecule is often very small, and often too these possess different PTMs. A related way to gauge the conformational diversity can be via a large-scale analysis of structures of proteins belonging to the same family [145]. The structural distribution observed in family members, which differ in sequence, is another reflection of a shift in the ensemble, as is of the proteins in different media [146]. Sequence divergence, media—water versus organic solvents—and molecular crowding are all changes in the environments, which redistribute the conformational ensemble. Conformational diversity of the native state modulates protein function [62, 147, 148] and may be revealed constructing and analyzing networks of evolutionarily coupled residues [149]. Other methods include mapping the conformational landscape of a dynamic enzyme by multitemperature and X-ray free electron laser (XFEL) crystallography [150]; however, these methods are unable to relate specific conformers to distinct functions. Another recent approach focused on proteins that switch folds via remodeling of secondary structure in response to a few mutations (evolved fold switchers) or cellular stimuli (extant fold switchers), both allosteric events. Putative extant fold switchers with only one solved conformation were identified by incorrect secondary structure predictions and likely independent folding cooperativity, resulting in an estimate that 0.5%–4% of PDB proteins switch folds [41]. A method that identifies functionally interacting mutations in both extant and reconstructed ancestral sequences models pairwise functional dependencies and higher-order interactions that enable evolution of new protein functions. The results reveal that function-preserving mutation dependencies are frequently from structural contacts, whereas gain-of-function mutation dependencies are most commonly between residues distal in protein structure [39].

## Conclusions: The significance of the conformational-level linkage

The “second molecular biology revolution” [2] calls for a new view of the genotype–phenotype dogma. The genotype does not encode only one state; it encodes ensembles of states. It is the ensembles that enable proteins to fulfill their functions; and thus, they are the ones that link genotype to phenotype. This new view of the genotype–phenotype association is not mere semantics. Its significance lies in deeper understanding of the connection of the disease phenotype with the genetic change and in providing the structural basis for disease-treating decisions [77]. Healthcare decisions are largely based on associating an observed disease phenotype with the genetic landscape, which is obtained through statistical analyses [151]. The conformational ensemble linkage can provide the mechanism of the mutation, point to cooperative “latent driver” mutations, and help in making predictions in precision medicine. Latent driver mutations behave as passengers; however, coupled with other emerging mutations, they may drive disease or phenotypic change [98]. Latent drivers are somatic mutations, emerging at any time during the individual life span. Eventually, the genetics of diseases is mediated by ensembles.

Classically, phenotype is what is observed; genotype is the genetic makeup serving as blueprint for protein expression and shape. Recently, the polycistronic nature of human genes has also been shown to be critical to understanding the genotype–phenotype relationship, and the addition of alternative open reading frames in databases argued to help relate phenotypes and genotypes [152]. However, today we also understand that a genotype is expressed not by a single protein shape but by a large number of shapes, some of which defining altered phenotypes. Key factors include the conformational heterogeneity, the populations, and the environment. The old view of the genotype–phenotype association is unable to explain how different combinations of mutations in the same gene lead to altered phenotypic changes, as in the case of the deer mouse above. Current concepts explain that all conformations preexist, and an altered pattern of mutations shifts the equilibrium of the conformational ensemble, which now displays changed characteristics. We believe that it is time to broadly link the age-old phenotype/genotype view. We now know: their association is mediated by the statistical distribution of the ensemble of states.

So why is an ensemble view of a genotype–phenotype linkage important? Why update our perspective when current approaches have been working? First, it allows understanding of the physicochemical basis of observations. Second, the advent of the so-called precision medicine, in which treatments are envisioned to be tailored to a person’s genetic profile, argues for a need to go beyond the multifactorial statistics. The free energy landscape of a protein can explain, and with time we hope quantify and predict [119, 153–155], how specific mutational combinations in an individual would alter the interactions of the protein and thus its cellular network and the prescribed drug regime. Conformational diversity of the native state can modulate protein function, with different ligands shifting the conformational equilibrium through their binding to highest-affinity conformers [147]. Conformational ensembles affect emerging functions and bear on enzyme and antibody promiscuity, signaling, protein–protein recognition, and preponderance of disease [156]. It is becoming increasingly clear that statistics based on combinations of multiple variables may not be enough to achieve this aim. Finally, third, creativity and innovation require in-depth understanding.
